# Study on optimization and evaluation system of traditional Chinese medicine rehabilitation program for swallowing disorder after stroke

**DOI:** 10.1097/MD.0000000000025731

**Published:** 2021-05-14

**Authors:** Qiang Tang, Biying Liang, Runyu Liang, Shiqiang Zhang, Luwen Zhu

**Affiliations:** a Rehabilitation Center; b Brain Function and Neurorehabilitation Laboratory, Second Affiliated Hospital of Heilongjiang University of Chinese Medicine, Nangang District; c Graduate School, Heilongjiang University of Chinese Medicine, Xiangfang District, Harbin, Heilongjiang, China.

**Keywords:** acupuncture, controlled trials, multicenter, randomized, stroke

## Abstract

**Background:**

Acupuncture has a long history in China and is currently widely used in clinical practice. However, there is no large sample data confirming the effectiveness of acupuncture in treating stroke and its sequelae. This article presents a study protocol for a multicenter, randomized, controlled trial for evaluating the efficacy of acupuncture in treating post-stroke dysphagia.

**Methods/design:**

A randomized controlled trial will be conducted in three hospitals in Heilongjiang, Changchun, and Beijing. A total of 252 patients with post-stroke swallowing ability will be randomly divided into two groups; specifically, the experimental group, which will receive acupuncture treatment, and the control group, which will undergo rehabilitation training for 2 weeks. The main results will be evaluated using the standard swallowing function scale, videofluoroscopic swallowing examination, and functional magnetic resonance imaging. The secondary observation indexes will include surface electromyography signal analysis and the modified Barthel index. Measurements will be obtained before treatment, as well as 1 week, 2 weeks, and 1 month after treatment.

**Discussion:**

This trial could clarify the effectiveness of acupuncture in stroke; moreover, it will determine whether acupuncture can improve swallowing function.

**Trial registration:**

Chinese Clinical Trial Registry (ChiCTR), ChiCTR2000030994.

## 1 Introduction

Stroke, which is also known as cerebral apoplexy, is characterized by high morbidity, mortality, recurrence, and disability rates; moreover, it has the second-highest mortality rate worldwide.^[1,2]^ The increasing stroke incidence in the young population is a significant public health issue. Moreover, the stroke burden has been increased by general population growth and the increasingly aging population.^[3]^

Stroke can cause several sequelae, including motor dysfunction, sensory dysfunction, cognitive function, and swallowing dysfunction. Approximately 65% of patients with acute stroke present dysphagia.^[4]^ Post-stroke damage to the brain, cerebellum, or brain stem can impair swallowing function.^[5]^ Swallowing difficulties cause several problems, including aspiration and malnutrition, which can cause further complications. Among them, pneumonia is the main mortality cause in patients with stroke; moreover, there is a need to address socioeconomic issues, such as long-term medical expenses.^[6,7]^

There has been rapid development and progress in modern rehabilitation technology, swallowing training, transcranial magnetic stimulation, electrical stimulation, and other methods, which allow significant improvements in post-stroke swallowing disorders. Acupuncture is an important treatment method in traditional Chinese medicine for rehabilitation. It has a definite effect, simple and easy operation, and less toxicity and side effects. It is a common clinical method for stroke treatment, including scalp acupuncture, nape acupuncture, tongue acupuncture, body acupuncture, and other needle methods. Currently, there have been numerous acupuncture studies on stroke treatment, including post-stroke swallowing dysfunction. Numerous clinical reports have indicated that acupuncture can significantly improve swallowing function.^[8,9]^ However, there remains no high-quality and effective evidence clearly showing acupuncture-mediated recovery of stroke and swallowing disorders.^[10]^

Consequently, the proposed study will use commonly used rating scales, surface electromyography (sEMG) signals, and functional magnetic resonance imaging (fMRI) to examine the acupuncture efficacy in the treatment of swallowing dysfunction in patients with stroke.

## 2 Methods/design

### 2.1 Objectives

The study objective is to formulate a TCM acupuncture rehabilitation program for post-stroke dysphagia (oral and pharyngeal stages). The central regulation mechanism underlying dysphagia treatment using TCM rehabilitation will be analyzed from the perspective of post-treatment brain function changes using resting fMRI and self-comparative analyses.

### 2.2 Study design

**Figure 1:**
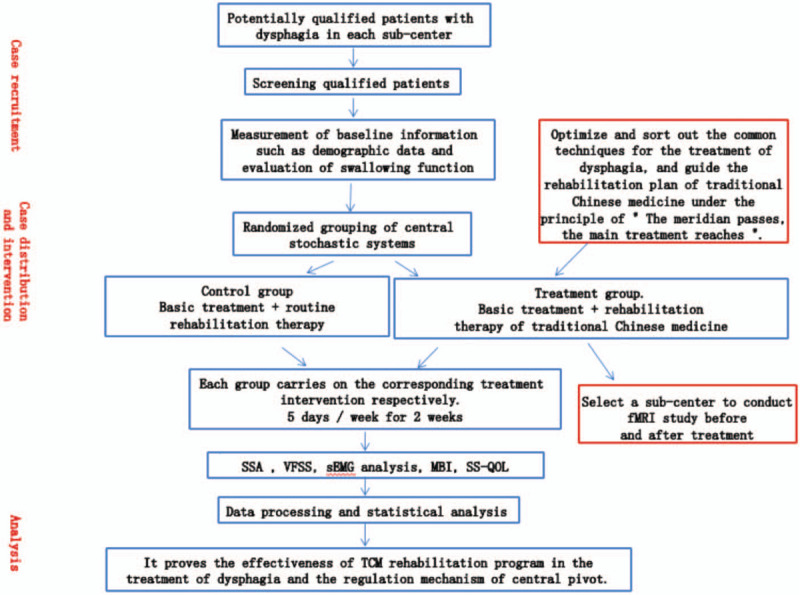
Present the technology roadmap.

This will be a multicenter, randomized controlled study conducted at the Second Affiliated Hospital of Heilongjiang University of Traditional Chinese Medicine, Changchun University of Traditional Chinese Medicine, and China Rehabilitation Research Center from December 1, 2018, to December 31, 2021. Patients with post-stroke dysphagia who meet the inclusion criteria will be divided into the experimental and control groups using a central randomization system. The experimental and control groups will receive acupuncture treatment and conventional rehabilitation training, respectively. The primary results will be obtained using the standardized swallowing assessment (SSA), videofluoroscopic swallowing study (VFSS), and fMRI. The secondary observation indicators will include surface electromyography (sEMG) analysis and modified Barthel index (MBI). The follow-up indicators include stroke-specific quality of life scale (SS-QOL) and the SSA. Measurements will be obtained before treatment, as well as 1 week, 2 weeks, and 1 month after treatment. fMRI will be only performed in the subject unit. Forty-two participants will undergo pre- and post-treatment fMRI assessment to analyze the central regulatory mechanism underlying swallowing function remodeling in the rehabilitation program of TCM. Figure 1 and Table 1 present the technology roadmap and clinical observation flow charts, respectively.

**Table 1:** Clinical observation flow charts.

Research stage	Selection period	Treatment period		Follow-up period
Evaluation times	First time	Second	Third time	Fourth
Evaluation time point	1 and 0 days before treatment	1 week after intervention	2 weeks after intervention	1 month after the end of the intervention
Inclusion criteria	•			
Exclusion criteria	•			
Informed consent form	•			
Online random grouping	•			
General assessment				
General information	•			
Current medical history/allergic history	•			
Past history/complications	•			
Evaluation of the curative effect index				
VFSS	•		•	
SSA	•	•	•	•
Surface electromyography signal	•	•	•	
Improved Barthel index	•	•	•	
SS-QOL				•
Security assessment				
Life indication	•	•	•	•
Other assessments				
Adverse reactions/events		•	•	
Recurrence situation				•
Treatment compliance			•	

SSA = standardized swallowing assessment, SS-QOL = stroke-specific quality of life scale, VFSS = videofluoroscopic swallowing examination.

### 2.3 Diagnostic criteria

The included participants will meet the following Western Medicine and TCM diagnostic criteria.

#### 2.3.1 Western medicine diagnostic criteria

Regarding the diagnostic criteria for cerebral infarction/cerebral hemorrhage, a confirmed diagnosis will be obtained through brain computed tomography (CT) or MRI, with reference to the Chinese guidelines for diagnosis and treatment of acute ischemic stroke and cerebral hemorrhage, at the Cerebrovascular Disease Section in Chinese Neurology Branch of Medical Association in 2014. The diagnosis for dysphagia will refer to the diagnostic criteria for post-stroke dysphagia rectified during the sixth National Academic Conference on Cerebrovascular Disease.

#### 2.3.2 TCM diagnostic criteria

Regarding the diagnostic criteria for cerebral infarction/cerebral hemorrhage, a confirmed diagnosis will be obtained through brain CT or MRI, with reference to the management guidelines for common diseases in internal medicine of TCM (ZYYXH/T19-2008). Dysphagia diagnosis will refer to the diagnostic criteria for post-stroke dysphagia rectified during the sixth National Academic Conference on Cerebrovascular Disease.

### 2.4 Inclusive criteria

The inclusion criteria will be as follows: clinical diagnosis of first-episode unilateral cerebral hemisphere infarction or cerebral hemorrhage, with confirmation using CT or MRI; meeting the diagnostic criteria for dysphagia; showing abnormal expressions of the oral and pharynx stages upon VFSS evaluation; having stroke and dysphagia duration within 3 months; being conscious with stable vital signs and a mini-mental state examination score of ≥ 21; age 45 to 75 years; and having provided informed consent.

### 2.5 Exclusion criteria

The exclusion criteria will be as follows: having a non-unilateral lesion; having a history of stroke, cerebral tumor, brain impairment, and other neuropsychiatric problems; having a history of dysphagia; having other cognitive disorders, aphasia, or serious cardiopulmonary and renal complications; and being unable to cooperate with examination and treatment.

### 2.6 The drop out of cases and disposal

The criteria for drop-out cases will be as follows: voluntary withdrawal from the study; being unsuitable for further assessment due to severe adverse reactions, severe complications, or condition deterioration; poor compliance that impedes the provision of consent for study participation; or incomplete treatment evaluation without rule compliance and with insufficient observation data.

Regarding the disposal of drop-out cases, researchers should adopt home visits, phone, letters, etc to communicate with the participants, record the last treatment time, and extensively perform evaluation. After obtaining the drop-out reasons, they will be added to the case report form. All observation data from drop-out cases will be collected for analysis by the project teams. The researcher should record details regarding drop-out information and enroll more than one treatment course to be included in the efficient statistics.

### 2.7 The discontinuity and management of cases

Participants shall be discontinued during the trials in case of occurrence of serious adverse reactions, severe complications, or condition deterioration. Moreover, the clinical trials will be suspended according to the doctors’ judgment, if required.

Regarding management of discontinued cases, the doctor will take some relevant clinical disposals after discontinuation. Subsequently, the doctor will perform a detailed investigation of the reasons and retain the obtained observation data. The last obtained result will be used as the final result and included in the analysis of efficacy and adverse reactions.

### 2.8 Case exclusion criteria

These will include cases with violation of inclusion or exclusion criteria; patients included in the wrong treatment group; and patients who withdraw from the trials, use prohibited drugs or treatments in combination, and with insufficient data.

### 2.9 Interventions

#### 2.9.1 Source and distribution of research objects

The patients will be randomly divided into the experimental and control groups (both n = 126). Eighty-four cases will be obtained in the Second Affiliated Hospital of Heilongjiang University of Traditional Chinese Medicine, Changchun University of Chinese Medicine, and China Rehabilitation Research Center.

#### 2.9.2 Treatment

The experimental group will undergo acupuncture at the appropriate neck acupoints as follows:

Nape cluster acupuncture treatment of the oral stage will employ the bilateral Fengchi, Gongxue, swallowing 2, Jiachengjiang, Xiaguan, Yingxiang, Lianquan, Waijinjin, Waiyuye, and Tongzhong acupoints.

Nape cluster acupuncture treatment of the pharynx stage will employ the bilateral Fengchi, GongXue, swallowing 2, Tiyan, Zhiqiang, swallowing 1, Phonation, Lianquan, Zhifanliu, Waijinjin, Waiyuye and Tongzhong acupoints.

The acupoints and acupuncture methods are described below. The Fengchi acupoint is at the same level as the Fengfu point, at the depression between the trapezius and sternocleidomastoid muscles. The needle tip will be slightly tilted downward and inserted 2 cm deep into the Adam's apple. The GongXue point is 2 cm below the FengChi point and flushes with the lower lip. The needle will be inserted 2 cm deep into the opposite lip. The swallowing 2 point is located at the flat chin lip groove and posterior edge of the sternocleidomastoid muscle. Here, the needle tip will be directly inserted approximately 1.5 to 2 cm into the opposite side and retained for 30 minutes. The JiaChengJiang point is on the face, 1 inch adjacent to the Chengjiang point. The XiaGuan point is ventral to the tragus and mandibular condyle at the depression formed by the zygomatic arch and mandibular notch. The Yingxiang point is located half an inch from the alar margin midpoint in the nasolabial groove.

The Lianquan point is located at the hyoid depression above the Adam's apple, with the needle being inserted 3 cm into the tongue base, twisted for 10 to 15 s, and then removed. The Waijinjin and Waiyuye acupoints are 1 cm on each side of the Lianquan point, with the needle being inserted 2 to 3 cm into the tongue base, twisted for 10 s, and then removed. The Tongzhong point (Juquan point) is at the middle of the tongue, with the needle being inserted 0.1 cm into the tongue and then removed. For the Fengchi, Gongxue, Jiachengjiang, Xiaguan, and Yingxiang acupoints, the needles will be retained in the insertion point for 30 min.

The Tiyan point, the needle will be directly inserted forward and downward at 1 to 1.5 cm at the depression in front of the mastoid and the lower earlobe edge. The Zhiqiang point is located between the hyoid bone and thyroid cartilage notch, with the needle being directly inserted approximately 0.3 to 0.5 cm, twisted for 10 to 15 s, and then removed. The swallowing 1 point is located at 0.5 cm adjacent to the midline between the hyoid bone and laryngeal node, with the needle being inserted approximately 0.5 cm laterally along the skin, twisted for 15 s, and then removed. The phonation point is located 0.5 cm below the midline of the Adam's apple; specifically, between the thyroid and cricoid cartilages. The needle will be inserted approximately 0.5 cm along the skin, twisted for 10 to 15 s, and then removed. The Zhifanliu point is located 1 cm behind the pronunciation point, the posterior edge of the cricoid cartilage, and the cricopharyngeal muscle. Regarding operation, the needle will be obliquely pierced 0.5 cm inward along the skin and removed after twisting for 10 to 15 s.

The control group will undergo swallowing function rehabilitation training. A tongue muscle rehabilitation device will be used to perform tongue muscle activities in the directions of front and back, up and down, left and right, oblique down, etc. Moreover, the patients will perform training involving mouth opening and closing, chewing, frowning, smiling, cheek puffing, and the Mendelssohn technique to promote swallowing. The lingual and soft palates will be stimulated using ice and acid.

##### 2.9.2.1 Dysphagia in the oral stage

1. Jaw, face, and cheek exercises
  The participant's mouth will be maximally opened, held for 5 s, relaxed for 3 s, and repeated 5 times. Moreover, the participants will close the lips, puff their cheeks, hold for 5 s, relax for 3 s, and repeat 5 times. Air will be rapidly moved around the left and right cheeks by puffing the cheeks 10 times. The participants will bare their teeth for 5 s for five sessions with 3-s relaxation intervals. Moreover, the participants will purse their lips for 5 s, relax for 3 s, and repeat the procedure five times. Finally, they will perform chewing movements ten times with 20-s intervals in between.
2. Lip exercises
  The participants will clench their teeth and pronounce Yi, hold for 5 s, and relax for 3 s for a total of five sessions. Moreover, the participants will purse their lips and pronounce Wu, hold for 5 s, and relax for 3 s for a total of five sessions. Next, the participants will alternately pronounce Yi and Wu for 3 s each and repeat the pronunciation twice in each session. The participants will complete three sessions and relax for 5 s. With tongue depressors on both lips, the participants will forcefully pull out the tongue depressor, resist with closed lips, hold for 5 s, and relax for 2 s, with the procedure being repeated five times. Regarding the blowing exercise, the participants will blow five times and breathe evenly for two cycles after each expiration, with a 20-s interval break.
3. Tongue exercises
  The participants will remove the tongue as far as possible out of the mouth, hold for 5 s, and retract and relax for 3 s, with this procedure being repeated ten times. Moreover, the participants will lift the tongue to the back of the front teeth, hold for 5 s, and relax for 3 s, with this procedure being repeated ten times. Furthermore, in each session, the participants will extend the tongue tip to the left and right lip angle for 2 s, with this procedure being repeated three times. The participants completed three sessions and relaxed for 5 s. Moreover, the participants will lick their lips with the tongue tip ten times with 20-s break intervals.
4. Velopharyngeal closure exercises
  An ice cotton stick will be used to stimulate the palatopharyngeal arch; simultaneously, the participants will pronounce “a” thrice, relax for 3 s, and repeat the procedure five times with 10-s break intervals.
5. Respiratory exercises
  The participant will breathe deeply by the nose while lifting the shoulders, and subsequently breathe out and relax. Each session will involve two respiratory cycles. The participants will complete three sessions with even breathing and a 5-s rest after each session. A 10-s break will be taken.
6. Mendelsohn's technique
  Here, the patients will be instructed to swallow empty air. The therapist will push the patient's throat using the hand to promote swallowing and the procedure will be repeated five times, with a 10-s break.

##### 2.9.2.2 Dysphagia in the pharyngeal stage

1. Tongue training
  The participant will extend the tongue from the mouth as much as possible, maintain it there for 5 s, and then retract and relax for 3 s, with the procedure being repeated 10 times. Moreover, they will open the mouth, lift the tongue to the back of the front teeth, maintain it there for 5 s, relax for 3 s, with the procedure being repeated 10 times. Additionally, in each session, the participants will extend the tongue tip to the left and then the right lip corner thrice for 2 s. Further, the participants will complete three sessions with 5-second relaxation breaks. Finally, the participants will lick the lips with the tongue tip and repeat 10 times, with a 10-s break.
2. Velopharyngeal closure training:
  To stimulate the velopharyngeal arch with an ice cotton swab will be used, and participant will simultaneously be instructed make a sound three times, relax for 3 s, repeat five times, and hold a break for 10 s.
3. Breathing training:
  The patients will be instructed to deeply inhale through the nose while lifting the shoulders; subsequently, they will shrink the mouth, exhale, and relax. Each session will be comprised of two breathing cycles. The participants will complete three sessions while breathing and resting for 5 s after each session. A 10-s break will be provided in between.
4. Mendelssohn manipulation
  The patient will swallow empty air, with the therapist pushing the patient's throat using the hands to promote swallowing. The procedure will be repeated five times with a 10-s break.
5. Direct feeding training.
  We will adopt empty, interactive, and side swallowing methods using viscous and uniform food for feeding training.

### 2.10 Outcome assessment

#### 2.10.1 Evaluation indexes

The primary outcome indicators will be the SSA and VFSS, while the secondary outcome indicators will be the sEMG signal analysis and MBI. The common evaluation index will be the SS-QOL while the follow-up indicators will be the SS-QOL and SSA.

#### 2.10.2 Evaluation time

Measurements will be obtained before the intervention, 1 week after intervention, and 2 weeks after intervention. The follow-up time point will be 1 month after the end of the intervention.

### 2.11 Ethical considerations

After the research plan is formulated by the project undertaking unit and approved by experts, it will be reported to the ethics committee of the project undertaking unit for approval. Each participating unit will report to the ethics committee to review its feasibility. Before study enrollment, the patients will be informed regarding the research background, purpose, benefits, and risks. The patients will be enrolled after providing informed consent. The adherence of the subjects was improved by scientific informed consent, active health education, humanistic care and long-term follow-up plan,.

### 2.12 Quality control

To ensure the research quality and data validity, we will conduct unified and standardized training for the study researchers, formulate clinical research SOPs, and ensure that the clinical research consistently follows the operating procedures. Regular monitoring will be used to ensure the study's authenticity and reliability.

### 2.13 Data management

To establish a database, all data will be double-entered using computer software to compile a data entry program. The data manager will ensure the completeness and integrity of the data in the case report form report entered into the computer. The data will be blindly reviewed; moreover, after correct confirmation, the data will be locked by the main researchers and statistical analysts.

### 2.14 Randomization and allocation concealment

The main researchers of each unit will be assigned different user names and passwords to establish a central random system and data collection system. Before treatment, the patient information will be entered into the central random system; moreover, the patient will be screened according to the inclusion and exclusion criteria. Patients who meet the criteria will be randomly divided into the control and experimental groups.

### 2.15 Blinding

In this study, due to the specificity of clinical acupuncture research, it is difficult to implement blind method. But it can be assessed by people who are not involved. We plan to assign specialized doctors from rehab centers as reviewers, and they will not be told how the subjects are grouped.

### 2.16 Sample size calculation

Referring to Liu Zhishun's “clinical study on acupuncture treatment of chronic moderate and severe dysphagia in stroke,” the effective rate of traditional rehabilitation is 80%, the estimated effective rate is 93.3%. According to the preset parameters, the effectiveness of the two groups were calculated with PASS 8.0 software, and 97 samples were needed for each group. At the same time, abscission and elimination were considered. The abscission rate was 30% and each group needed 126 samples. Therefore, 252 subjects were expected to be included in this study.

### 2.17 Statistical analysis

A third-party medical statistics professional (Guangdong Provincial Hospital of Traditional Chinese Medicine) will perform statistical analysis. After the subject research plan and case report form are reviewed by experts, a third party will formulate a statistical analysis plan and determine the statistical software to use.

1. Full Analysis Data Set (FAS): A total of 252 random numbers were designed in this study to exclude subjects in a minimum and reasonable manner according to the principle of intentionality analysis. This data set includes all subjects receiving treatment. Missing data for key indicators are carried forward to the closest observation (LOCF).
2. Per-Protocol Data Set: those who have good compliance (who have received at least 2/3 or more of the courses of treatment), whose main indicators of observation are not missing, who have basically not violated the Protocol, and who meet the inclusion criteria without any exclusion indicator constitute the current PP population. Missing data in this data set is not carried forward, but is treated as missing data.

## 3 Discussion

Dysphagia refers to swallowing dysfunction.^[11]^ Clinical patients are commonly in the oral, pharyngeal, or both stages. The mouth stage is the chewing food process to form a bolus, with the tongue moving the bolus back and forth before entering the pharynx. Dysfunction involves weakness of the facial muscles, including the chewing, labial, and tongue muscles, which impedes food bolus formation and smooth delivery to the pharynx. The pharyngeal stage refers to the process of the food bolus entering the oropharynx into the esophagus. This stage involves abnormal pharyngeal muscle and laryngeal function, which often causes aspiration, pharynx retention, and hoarseness.^[12]^

The SSA is divided into three parts: clinical examination, observation of patients swallowing 5 mL of water, and observation of patients swallowing 60 mL of water. It has a total score of 46, with the lowest possible score being 17. A higher cumulative score of each item indicates worse swallowing function. Some studies have reported that SSA is more feasible and suitable for evaluating dysphagia.^[13]^ The SSA includes 10 items divided into four levels: independent, less dependent, moderately dependent, and completely dependent. The VFSS allows dynamic assessment of the swallowing function of the mouth, pharynx, and upper esophagus using TV fluoroscopy. The VFSS is considered the “gold standard” for evaluating swallowing function. It is widely used for the comprehensive evaluation of the patients’ oral, pharyngeal, and esophageal swallowing conditions.^[14]^ The sEMG allows evaluation of the physiological process of swallowing. It is a simple and reliable screening that allows preliminary identification of dysphagia and has advantages of accuracy, rapidity, and non-invasiveness.^[15]^ fMRI is a safe, non-invasive, and non-radiation imaging method with high temporal and spatial resolution. Anatomical and functional images can be simultaneously obtained in a single imaging session.^[16]^

Acupuncture is commonly applied in stroke rehabilitation; however, there remains no high-quality evidence regarding acupuncture efficacy. There are numerous acupuncture types, including scalp, body, nape, and latch acupunctures, as well as awn needles, which are all used for the treatment of post-stroke swallowing dysfunction. Neck acupuncture can directly act on the throat, as well as stimulate hypoglossal and recurrent laryngeal nerves. Fengchi and other acupoints can stimulate the occipital artery, occipital vein, vertebral artery, and vertebral vein, as well as promote blood circulation.^[17]^ This large-scale, multicenter, randomized controlled trial will employ neck acupuncture to assess its treatment effect on post-stroke swallowing dysfunction.

## Author contributions

**Conceptualization:** Qiang Tang, BiYing Liang, Luwen Zhu.

**Investigation:** Qiang Tang, BiYing Liang, Runyu Liang, Shiqiang Zhang.

**Supervision:** Luwen Zhu.

**Writing – original draft:** Qiang Tang, BiYing Liang.

**Writing – review & editing:** Qiang Tang, BiYing Liang.
